# Neutrophils in Leprosy

**DOI:** 10.3389/fimmu.2019.00495

**Published:** 2019-03-19

**Authors:** Veronica Schmitz, Isabella Forasteiro Tavares, Patricia Pignataro, Alice de Miranda Machado, Fabiana dos Santos Pacheco, Jéssica Brandão dos Santos, Camila Oliveira da Silva, Euzenir Nunes Sarno

**Affiliations:** ^1^Leprosy Laboratory, Oswaldo Cruz Institute, FIOCRUZ, Rio de Janeiro, Brazil; ^2^Laboratory of Cellular Microbiology, Oswaldo Cruz Institute, FIOCRUZ, Rio de Janeiro, Brazil

**Keywords:** leprosy, *Mycobacterium leprae*, erythema nodosum leprosum, inflammation, neutrophils

## Abstract

Leprosy is an infectious disease caused by the intracellular bacillus *Mycobacterium leprae* that mainly affects the skin and peripheral nerves. One of the most intriguing aspects of leprosy is the diversity of its clinical forms. Paucibacillary patients are characterized as having less than five skin lesions and rare bacilli while the lesions in multibacillary patients are disseminated with voluminous bacilli. The chronic course of leprosy is often interrupted by acute episodes of an inflammatory immunological response classified as either reversal reaction or erythema nodosum leprosum (ENL). Although ENL is considered a neutrophilic immune-complex mediated condition, little is known about the direct role of neutrophils in ENL and leprosy disease overall. Recent studies have shown a renewed interest in neutrophilic biology. One of the most interesting recent discoveries was that the neutrophilic population is not homogeneous. Neutrophilic polarization leads to divergent phenotypes (e.g., a pro- and antitumor profile) that are dynamic subpopulations with distinct phenotypical and functional abilities. Moreover, there is emerging evidence indicating that neutrophils expressing CD64 favor systemic inflammation during ENL. In the present review, neutrophilic involvement in leprosy is discussed with a particular focus on ENL and the potential of neutrophils as clinical biomarkers and therapeutic targets.

## Introduction

Leprosy is a millennial disease that continues to adversely impact the public health systems of endemic countries. The most commonly affected sites are the dermis and the peripheral nerves. Permanent disabilities are the direct consequence of the neurological damage caused by the *Mycobacterium leprae* infection, especially when the damage is left untreated in its early stages. During 2017, 150 countries reported 210,671 new cases of leprosy at a detection rate of 2.77/100,000 (1).

Leprosy severity is determined by the regulation of cell-mediated immunity, ranging anywhere from mild, presenting with a single, well-demarcated lesion (termed *tuberculoid*: TT), to severe, involving widespread, poorly-demarcated, raised, or nodular lesions (termed *lepromatous lepromatous*: LL). Biopsies of TT lesions reveal well-developed granulomatous inflammation associated with the marked presence of Langerhans cells (CD1a^+^) and rare acid-fast bacilli. LL dermal lesions are characterized by the presence of numerous heavily-infected foamy macrophages, a sparse infiltrate of lymphoid cells, and the number of Langerhans cells is consistently low (2–4). The so-called borderline patients (BT, BB, and BL) are situated between the extremes of the TT and LL poles. These patients display a mixed unstable immune response whose characteristics are in accordance with their proximity to one pole or the other (5).

**Figure 1:**
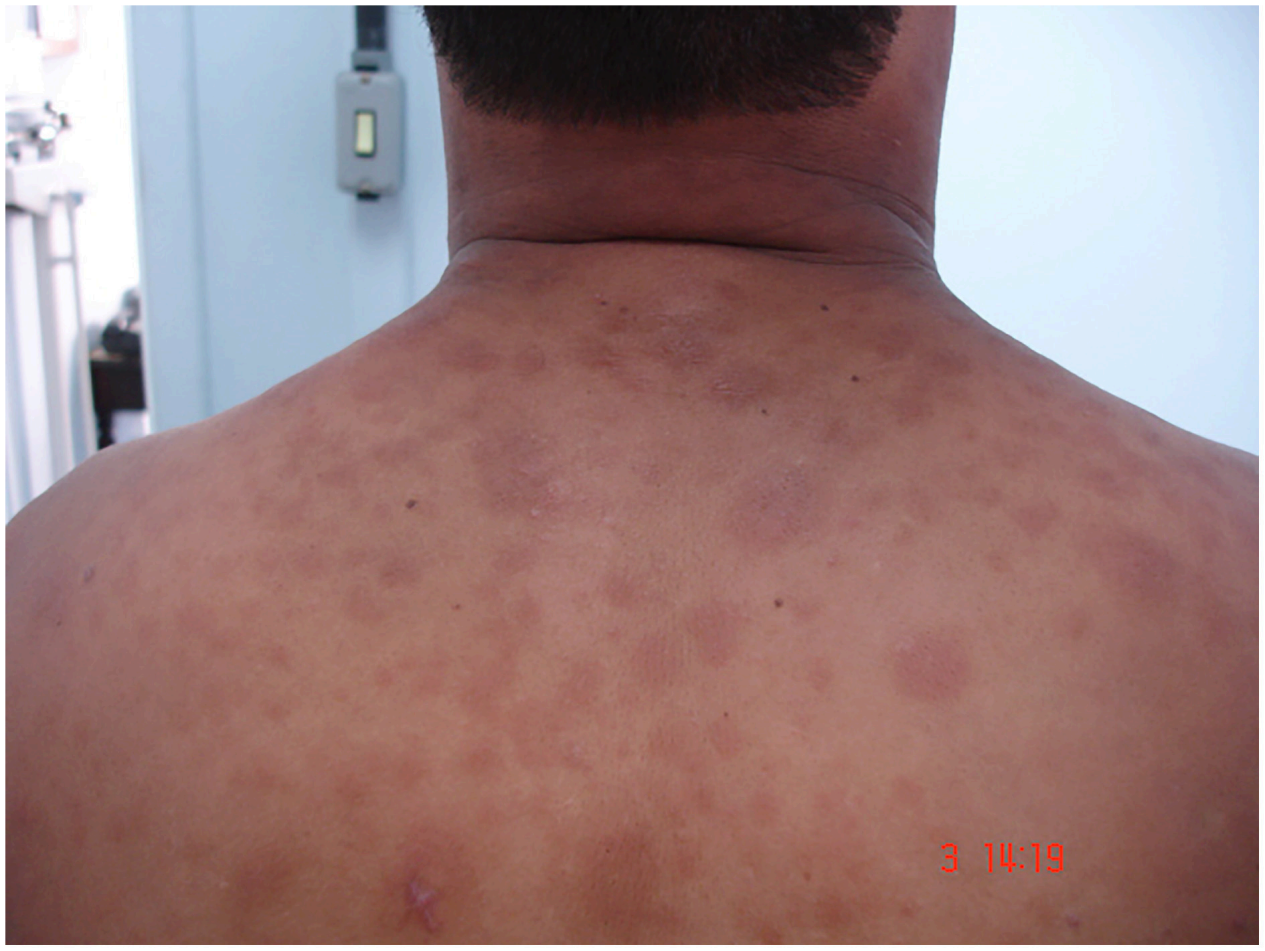
Skin lesions of ENL patient. Image from Leprosy Laboratory collection.

During disease evolution, 50% of LL and 5–10% of BL patients present a variety of dermatological inflammatory phenomena with systemic symptoms (6, 7), referred to as erythema nodosum leprosum (ENL, or Type 2 reaction). ENL together with reversal reaction are core aspects of leprosy that profoundly impact both the course of the disease and the development of nerve damage (8). Clinically, ENL patients demonstrate painful subcutaneous nodules on the apparently normal skin (Figure 1). More severe cases display systemic inflammation accompanied by neutrophilic leukocytosis, fever, and malaise similar to sepsis (9).

**Figure 2:**
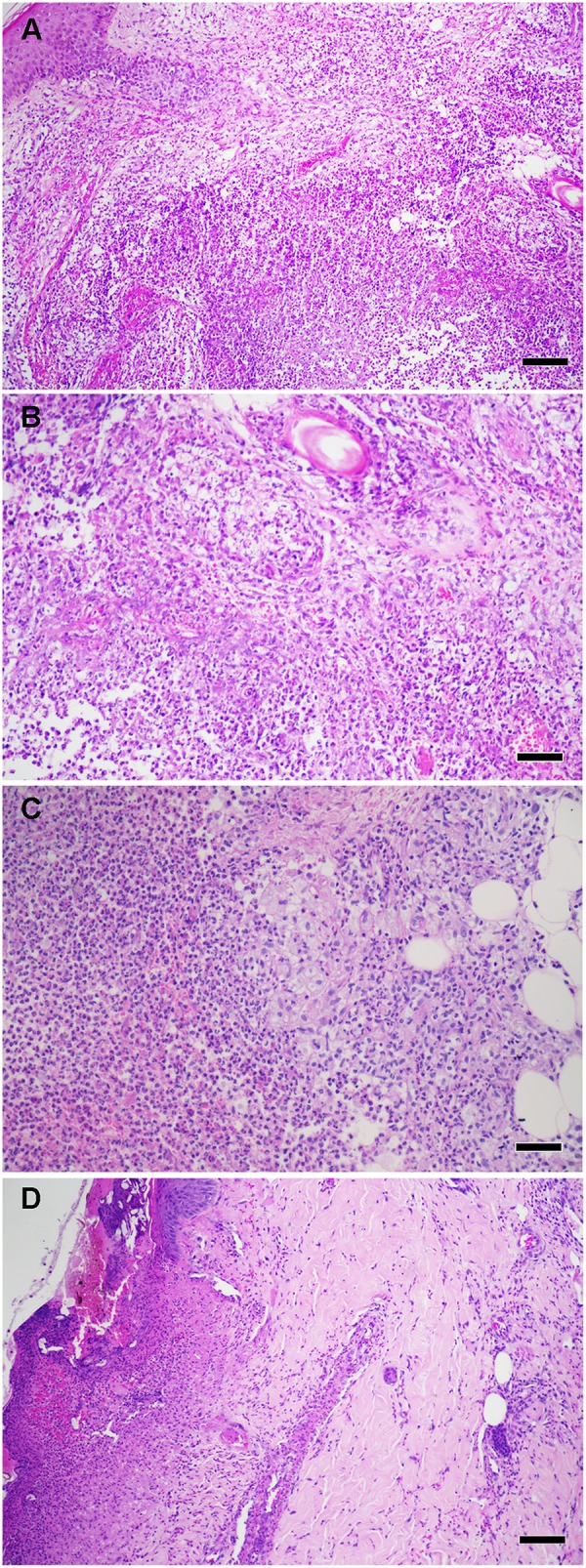
**(A)** Histopathological aspects of ENL skin lesions. **(B)** High power showing foamy macrophages and neutrophil infiltrate. **(C)** High power showing a collection of neutrophils (microabscess) in deep dermis. **(D)** Necrotizing ENL, Epidermal ulceration with vasculitis. Hematoxylin and eosin staining (scale bars: 100 μm). Images from Leprosy Laboratory collection.

Histologically, ENL lesion has prominent neutrophilic infiltrate mainly lodged inside the deep layers of the dermis and subcutaneous tissue superimposed on chronic multibacillary leprosy (Figure 2). A cluster of foamy macrophages containing fragmented bacilli and a high number of Langerhans cells in dermis and epidermis are usual (4, 10–14). Eosinophils, lymphocytes and plasmocytes are also found together with neutrophils. It seems that with the evolution of the ENL lesions, the number of lymphocytes and plasmocytes increases, while the number of neutrophils and eosinophils decreases (11, 15–18). Vascular abnormalities (endothelial swelling, edema, and angiogenesis) are consistently observed in acute stage of ENL lesions and reduced after anti-reactional treatment (11, 18–20). The ulcerated form, called necrotizing ENL, demonstrates similar, though more intense, histological findings and leukocytoclastic vasculitis is observed (17, 21, 22) (Figure 2).

Currently, ENL is often designed as a neutrophilic immune complex-mediated disease (23). The cause of ENL is eminently complex. Immune complex deposits have been implicated in the cutaneous lesions of ENL (13, 24). It is primarily driven by an aberrant dermal immune response that is modified by genetic susceptibility (25) and various environmental stimuli (e.g., pregnancy, lactation, puberty, intercurrent infections, vaccination, and psychological stress) (26). Elevated levels of tumor necrosis factor (TNF)-α and other pro-inflammatory cytokines have been associated with ENL episodes while, in the opposite direction, TNF suppression leads to clinical improvement (27, 28). Learning more about the factors that ultimately trigger and/or sustain ENL could lead to the identification of strategies to control and, most importantly, prevent the associated inflammation.

A case can be made that the role played by neutrophils in leprosy has been largely overshadowed by several studies dedicated to the macrophages and Schwann cells targeted by *M. leprae* (29). In the blood of multibacillary leprosy patients (LL and BL), neutrophils and monocytes are loaded with the bacilli (30) and their clearance will only effectively occurs after 2–3 months of multidrug therapy (31). Novel aspects of neutrophilic biology reported in recent papers strongly indicate that, in ENL, neutrophils are active and not neutral, thus providing new insights into their participation in the disease. In the present review, we tried to highlight some of the potential gaps in knowledge among neutrophils in leprosy. Our focus was on attempting to identify the possible ways neutrophils might contribute to ENL-linked systemic inflammation. As a final concern, the potential of these cells as clinical biomarkers and therapeutic targets was highlighted.

## Some Old and New Findings on Neutrophil Biology

Neutrophils have always been considered effector cells of innate immunity with a limited biosynthetic capacity. The primary role ascribed to these cells was as warriors against extracellular pathogens and in acute inflammation. These cells were classically characterized by their phagocytic ability, the release of lytic enzymes from their granules, and the production of reactive oxygen intermediates with a microbicidal potential. In the 1990s, however, this limited view was challenged by evidence that neutrophils actually survive much longer than initially believed (32) and have added ability to express genes encoding proinflammatory key mediators as components of the complement system, Fc receptors, chemokines, and cytokines (33).

Neutrophils are continuously generated in the bone marrow from its myeloid precursor. Daily production approximates 2 × 10^11^ cells. In humans, 50–70% of circulating leukocytes are neutrophils whereas, in mice, they range from 10 to 25%. This process is largely controlled by the granulocytic colony stimulating factor (G-CSF), produced in response to interleukin 17A (IL-17A). IL-17A is primarily synthesized by Th17 cells. But, innate immune cells, including γδ T cells, neutrophils, macrophages, innate lymphocyte cells (ILC), mast cells, and keratinocytes, have recently been found to be involved in IL-17 secretion (34). Other molecules—such as granulocytic–macrophage-colony stimulating factor (GM-CSF), IL-6, and KIT ligand (KITL, also known as KITLG)—likewise induce granulopoiesis. The production of this cytokine storm during the inflammatory responses results in overactive granulopoiesis and neutrophilia. During maturation, neutrophils undergo a number of stages referred to as either myeloblasts, pro-myelocytes, myelocytes, metamielocytes, band neutrophil, or, lastly, polymorphonuclear cells (segmented). Neutrophilic granules are formed sequentially during maturation of the pro-myeloid stage (35).

In the circulation, mature neutrophils have an average diameter of 7–10 μm, segmented nucleus, and enriched cytoplasmic granules and secretory vesicles. Three types of granules are formed during neutrophilic maturation, as follows: (i) azurophilic (or primary) containing myoloperoxidase (MPO); (ii) specific (or secondary) containing lactoferrin; and (iii) gelatinase (or tertiary) containing metalloproteinase 9 (MMP9, or gelatinase B). In humans, azurophilic granules are differentiated into defensin-poor and -rich ones (36).

Neutrophils have long been considered short, half-life cells in the circulation that normally survive approximately 1.5 h in mice and 8 h in humans (37, 38). Pillay et al. demonstrated that, under baseline conditions, the average life span of neutrophils in the circulation is 12.5 h in mice and 5.4 days in humans (37). During inflammation, neutrophils become activated and longevity increases, ensuring the presence of these cells at the inflammation site (32, 39). Endogenous products such as cytokines and growth factors together with bacterial products activate neutrophils. This increased half-life may allow neutrophils to perform more complex activities in the tissue. Examples may include: resolution of inflammation through the production of lipid mediators, modulation of the adaptive response, and reverse transmigration, which could involve the ability to exit the initial injury site and migrate to other tissues such as bone marrow (40).

Neutrophils eliminate pathogens through various intra- and extracellular mechanisms. When neutrophils encounter microorganisms, phagocytosis occurs followed by the formation of phagosomes. Microorganisms could be killed by NADPH oxygenase-dependent pathways (reactive oxygen species, ROS) or antimicrobial proteins (cathepsins, defensins, lactoferrin, and lysozyme) (35). These microbial proteins are released into either the phagosomes or the extracellular environment, acting against intra- or extracellular pathogens, respectively.

ROS production, i.e., oxidative burst, is considered a key component of the innate immune defense against bacterial and fungal infections (41). To the best of our knowledge the literature hasn't shown yet oxidative burst in leprosy neutrophils. However, oxidative stress was evaluated by measuring serum levels of malondialdehyde (MDA) and superoxide dismutase (SOD) activity and the results showed that leprosy patients present increased serum levels of MDA, MDA/SOD ratio together with a decreased SOD activity when compared to healthy controls suggesting oxidative stress in leprosy (42). In addition, it was demonstrated that the oxidative stress gradually increased along the spectrum from TT to LL (43).

An elegant strategy was used to measure the antimicrobial capacity of neutrophils to contain methicillin-resistant *Staphylococcus aureus* (MRSA). Leliefeld et al. (44) used a long-term neutrophil bacterial interaction in a 3D scaffold reminiscent of the *in vivo* environment. In such condition it was possible to evaluate the capacity for long-term intracellular containment of live bacteria. Neutrophils from chronic granulomatous disease (CGD) patients who lack a functional NADPH oxidase and healthy neutrophils under hypoxic conditions did not exhibit impaired bacterial containment and in that way this containment was independent of ROS (44). Conversely, failure of the phagosomal acidification led to impaired intracellular containment of MRSA (44).

Activated neutrophils have the capacity to eliminate extracellular microorganisms after releasing neutrophilic extracellular traps (NETs) (45). NETs are composed of nuclear DNA in association with histones and granular proteins such as antimicrobial proteins and enzymes (MPO and neutrophil elastase). The functions of NETs include immobilizing pathogens to prevent them from disseminating and facilitating the subsequent phagocytosis of trapped microorganisms. Many pathogens, namely viruses, bacteria, parasites, and fungi can induce NETs. For this reason, the mechanisms for both the initiation and evasion of NETs by pathogens have been intensively studied (46). To our knowledge, no report exists in the literature regarding the ability or inability of *M. leprae* to induce NETs. In addition, there are two open questions: (1) Is there a connection between NET formation, neutrophil infiltration and ENL systemic inflammation? (2) Does the massive presence of neutrophils in the ENL lesions could generate necrotic areas? It is possible to observe a collection of neutrophils in ENL lesions forming a microabscess (Figure 2). The NETosis role in limiting the spread of necrotic tissues was demonstrated in acute abdominal inflammation where netting neutrophils create a barrier between necrotic and viables areas (47).

Although ROS production by neutrophils during infections is an important antimicrobial mechanism, the exacerbated production of ROS due to the massive involvement of neutrophils can lead to oxidative stress accompanied of cell death and necrosis (47). The large amounts of neutrophil-released enzymes could degrade cytokines but also modify glycan on the surrounding tissues (48). Alteration of glycan residues on IgG molecules is associated with high lupus disease activity (49). Moreover, the incomplete clearance of DNA-released material leads to systemic inflammation and autoantibody production (50).

Dias et al. (51) demonstrated that ENL patients displayed higher levels of human DNA–histone complexes than either BL/LL patients or healthy individuals. The increased levels of TLR-9 ligands and the TLR-9 *per se* in peripheral mononuclear cells was considered by the authors as a major innate immunity pathway activated during ENL (51). Meanwhile, the source of the DNA-histone complex has yet to be identified.

There is much evidence in support of the existence of neutrophilic subpopulations and their role in inflammation, infection, and tumor immunology. The neutrophils subsets have been characterized according to their phenotypic, functional, morphological, and physical characteristics under both homeostatic and pathophysiologic conditions (Table 1). A detailed description of all neutrophils subsets is beyond the scope of this work, but additional reviews can be found elsewhere (58). Nonetheless, it is not yet known whether these subpopulations are distinct subsets or rather represents the plastic development of their neutrophilic precursor.

**Table 1:** Studies of neutrophil subpopulations.

**Neutrophil subsets**	**Findings**	**References**
**MRSA MOUSE MODEL OF INFECTION**		
PMN-I (MRSA - resistant host)	CD49d^+^/CD11b^−^	(52)
	IL-12 and CCL3 production	
	Classically activated macrophages	
	Expression of TLR2, TLR4, TLR5, TLR8	
	Multi-lobular nucleus	
PMN-II (MRSA-sensitive hosts)	CD49d^−^/CD11b^+^	
	IL-10 and CCL2 production	
	Alternatively activated macrophages	
	Expression of TLR2, TLR4, TLR7, TLR9	
	Ring-shaped nucleus	
PMN-N (normal host)	CD49d^−^/CD11b^−^	
	No production of cytokines and chemokines	
	No effect on macrophage activation	
	Expression of TLR2, TLR4, TLR9	
	Round nucleus	
**HEALTHY VOLUNTEERS CHALLENGE WITH I.V. LPS**		
CD16^bright^/CD62L^dim^	Not found in healthy donors	(53)
	Hypersegmented neutrophils	(54)
	Rapid apoptosis rate (similar to normal)	(44)
	Higher expression of CD11b, CD11c and CD54	
	Inhibit T cell proliferation	
	Normal phagocytosis	
	Poor capacity to contain intracellular bacteria	
	Less chemotactic rate	
	Decreased adhesion	
CD16^dim^/CD62L^bright^	Not found in healthy donors	
	Banded neutrophils	
	Higher rate of survival	
	Higher NADPH oxidase activity	
	Higher acidification of phagosome	
	Contain intracellular bacteria	
	Enhanced adhesion	
	Higher chemotactic rate	
CD16^bright^/CD62L^brigh^	Phenotypically mature (normal)	
**TUMOR ASSOCIATED NEUTROPHILS (TANS)**		
N1	Pro-inflammatory properties	(55)
	TNF production	(56)
	High tumoral cytotoxicity	(57)
	High NET production	
	ICAM1^high^	
	Hypersegmented nucleus	
N2	Anti-inflammatory	
	High production of arginase	
	Immature-like or segmented nuclei	
	Proangiogenic profile	
	Higher production of MMP-9 and VEGF	

*MRSA, methicillin-resistant Staphylococcus aureus*.

Recruitment of leukocytes at a site of blood vessel growth is a crucial event for proper angiogenesis and subsequent tissue perfusion. Pro-angiogenic neutrophils CD11b^+^/GR-1^+^/CXCR4^high^ producing high levels of MMP9 were recruited into the tissue in response to VEGFA in a mouse model of non-vascularized transplant (59). These pro-angiogenic neutrophils have been shown to be essential in promoting neovascularization of transplanted pancreatic islands (59) and may be the same cells that are known to promote cancer cell survival (60).

Neutrophils with regulatory function were identified in several models. Lung neutrophils isolated from both bronchoalveolar lavage fluid and parenchyma of infected mice produced IL-10 and negatively regulating local lung inflammation during chronic phase of *M. tuberculosis* infection (61). Secreting IL-10 neutrophils reported in *Trypanosoma cruzi*-infected mice showed an IL-10-dependent suppressive phenotype *in vitro* inhibiting T-cell proliferation and IFN-γ production (62). However, these anti-inflammatory neutrophils may change into pro-inflammatory phenotypes (IL-10^low^/IL-12^high^) after interacting with Natural Killer T cells in a CD1d-dependent manner (63). It was showed that neutrophils (G-neutrophils) from G-CSF–treated human and mice donors could inhibit T cell activation both *in vitro* and *in vivo* in a model of experimental acute graft-versus-host disease (64, 65). The disease inhibition induced by G-Neutrophils is dependent on neutrophil IL-10 competence (66).

During infection with MRSA, distinct types of neutrophils have been identified in association with resistance and susceptibility to MRSA. Neutrophilic populations isolated from both resistant and susceptible MRSA, PMN-I, and PMN-II, respectively—were distinct from neutrophils isolated from healthy mice, PMN-N (Table 1). It is possible that these pro- and anti-inflammatory neutrophils may alter the course of the adaptive response by inducing M1 or M2 macrophages, respectively. It cannot be ruled out that these neutrophils may change their phenotypes during the course of inflammation to fit a particular aggressor and do not necessarily represent distinct lineages (52). To date, no work in the literature has identified any neutrophilic subpopulations in leprosy that might correlate with the TT leprosy-resistant or LL susceptible leprosy polar forms, TT vs. LL.

A myriad of neutrophilic subpopulations (CD16^bright^/ CD62L^dim^, CD16^dim^/ CD62L^bright^, CD16^bright^/CD62L^bright^) has been identified in the circulation of human volunteers receiving lipopolysaccharide in contrast to the number in untreated individuals (53, 54) (Table 1). The subset CD16^bright^/CD62L^dim^ hypersegmented neutrophils displayed normal phagocytosis associated with a remarkably poor capacity to contain bacteria intracellularly. This defect in bacterial containment was associated with failure of acidification in the phagosomal compartment. On the other hand, CD16^dim^/CD62L^bright^ banded neutrophils were the only neutrophil subset that adequately contained MRSA (44).

Fridlender et al. (55) were the first to describe the existence of additional neutrophilic subsets nominated N1 e N2. The former is pro-inflammatory and the latter, anti-inflammatory (55). Via a murine model of cancer, the authors demonstrated the presence of tumor associated neutrophils (TANs), characterized by differential activation and phenotypical states. Neutrophils with an N1 phenotype possess a hypersegmented nucleus with pro-inflammatory and antitumor properties due to increased tumoral cytotoxicity, high NETs production, high ICAM1 expression, and production of inflammatory cytokines and chemokines like TNF. On the other hand, the N2 phenotype plays an opposite role and is classified as immunosuppressive and pro-tumoral mostly due to the elevated production of arginase like G-MDCs. These neutrophils usually possess an immature nucleus although some works have described them as being segmented. Another interesting fact about N2 neutrophils is their proangiogenic profile, which is driven by their capacity to produce elevated levels of MMP-9 and VEGF (57). Furthermore, it has been shown that TGF-β plays a critical role in neutrophilic polarization as a result of its ability to induce plasticity between a N1 subset into a N2 profile (55, 56). Other factors, namely angiotensin-II and type I IFNs, have recently been to shown to promote N1/N2 polarization too (58). Beyond phenotypic differences, it became clear by way of the transcriptomic approach that the N1 and 2 subsets represent distinct populations with diverse transcriptional signatures (56).

Interestingly, a new neutrophil subset with different densities has been the focus of several research projects. Associated with disease severity in some inflammatory disorders, a subset of low-density neutrophils (LDN) that co-localizes with peripheral blood mononuclear cells (PBMC) after density gradient separation has been reported (67). It is also noteworthy that, in cancer, this population was found to increase within tumor growth and be characterized by a morphologically homogeneous population that may contain band and segmented neutrophils (68, 69). Even though the origin and role of this subpopulation remain somewhat nebulous, some works have reported that LDNs display diverse profiles. The analyses of PBMC preparations from patients with Systemic Lupus Erythematous reveals that LDNs have an activated phenotype. In this scenario, LDN produce higher levels of such pro-inflammatory mediators as type I IFNs, IFN-γ, and TNF and are capable of modulating endothelial cell functions and increasing vascular damage (70). In addition, they are more disposed to form NETs that favor the chronic inflammation and disease severity (71, 72). While LDN have also been detected in many other pathologies like sepsis, HIV infection, malaria and also in tuberculosis (73–76), increasing our understanding of their surface marker patterns, cytokine expression, transcription factor regulators, and other trademarks of activation is of prime importance. Despite the uptick of studies describing the diversity of neutrophilic subpopulations, their distinct origins and plastic capacity remain unknown. New data need to be put forward that corroborate the existence of neutrophilic subpopulation in leprosy.

Under certain physiological conditions, the death of circulating neutrophils takes place in the liver, spleen, and bone marrow. Observed in old neutrophils, increased CXCR4 expression helps to direct them back to the bone marrow and subsequent elimination. CXCR4 is also involved in the down regulation of the newly-formed neutrophilic release into the marrow (39). Some studies suggest that terminal neutrophilic trafficking inside the intestinal tract also takes place to help regulate the commensal flora (77). Both senescent neutrophils and those that die after fighting infection may also expire within the vasculature and be removed by Kupffer cells (resident liver macrophages) (78). Removal of neutrophils by both Kupffer and dendritic cells is mainly regulated by the IL-23/IL17/G-CSF axis. This cytokinetic axis stimulates neutrophilic production in the bone marrow and is down regulated by liver X receptor (LXR) (79). The mechanisms involved in the clearance of NETotic cells are not yet known. On the other hand, clearance of apoptotic cells is well-studied (50). Different cell types participates in the uptake of apoptotic bodies by employing different types of receptors. This process can be amplified or suppressed by different types of plasma proteins (80). The complement system, pentraxins, and collectins have been implicated in apoptotic cell clearance in circulation or in injured tissue (81, 82).

## Neutrophils in Leprosy

A number of studies in the 1970's addressed the neutrophilic functions in the different forms of leprosy (83–86). These studies used the nitro blue tetrazolium that measures neutrophilic activation through reduction of *in vitro*. Goihman-Yahr et al. (83, 85) found that, during reaction, there is spontaneous neutrophilic activation not witnessed in LL leprosy (83, 85). Moreover, neutrophils are equally well-activated by endotoxin and *M. leprae in vitro* (87) in all forms of leprosy. In sharp contrast, Sher et al. (86) found no spontaneous activation rate in neutrophils of TT, LL, or ENL patients. Even so, ENL sera activated neutrophils of healthy donors *in vitro*, suggesting that ENL sera contain a neutrophilic activation inductor (86). Another parameter assessed of neutrophilic activation was cell motility that was measured by three different assays (random migration, chemotaxis, and chemokinesis), all of which were defective in LL neutrophils whether in the absence or presence of ENL (86). Drutz et al. (88) reported no important differences among TT and LL patients and normal control subjects in the bactericidal and fungicidal functions of their phagocytic cells, including monocytes, macrophages, and neutrophils (88).

Circulating neutrophils from leprosy patients are loaded with *M. leprae* (30, 31) and there is apparently no sign of systemic inflammation. It remains unclear whether neutrophils are capable of killing the bacilli. Our group has reported that neutrophils isolated from LL patients with or without ENL released TNF and IL-8 subsequent to stimulation with *M. leprae ex vivo* (89). Besides, the apoptotic rate of ENL neutrophils is higher in comparation to that found in BL/LL patients and healthy volunteers (89). It has been previously demonstrated that apoptotic neutrophils infected or not with *M. tuberculosis* trigger a proinflammatory response in *M. tuberculosis*-infected macrophages through a caspase-1- and IL-1β-dependent mechanism (90). The biological responses of macrophages included an enhanced production of proinflammatory cytokines as well as an enhanced capacity to control the intracellular growth of *M. tuberculosis*. The interaction of apoptotic neutrophils and macrophages in leprosy has yet to be determined with certainty.

**Figure 3:**
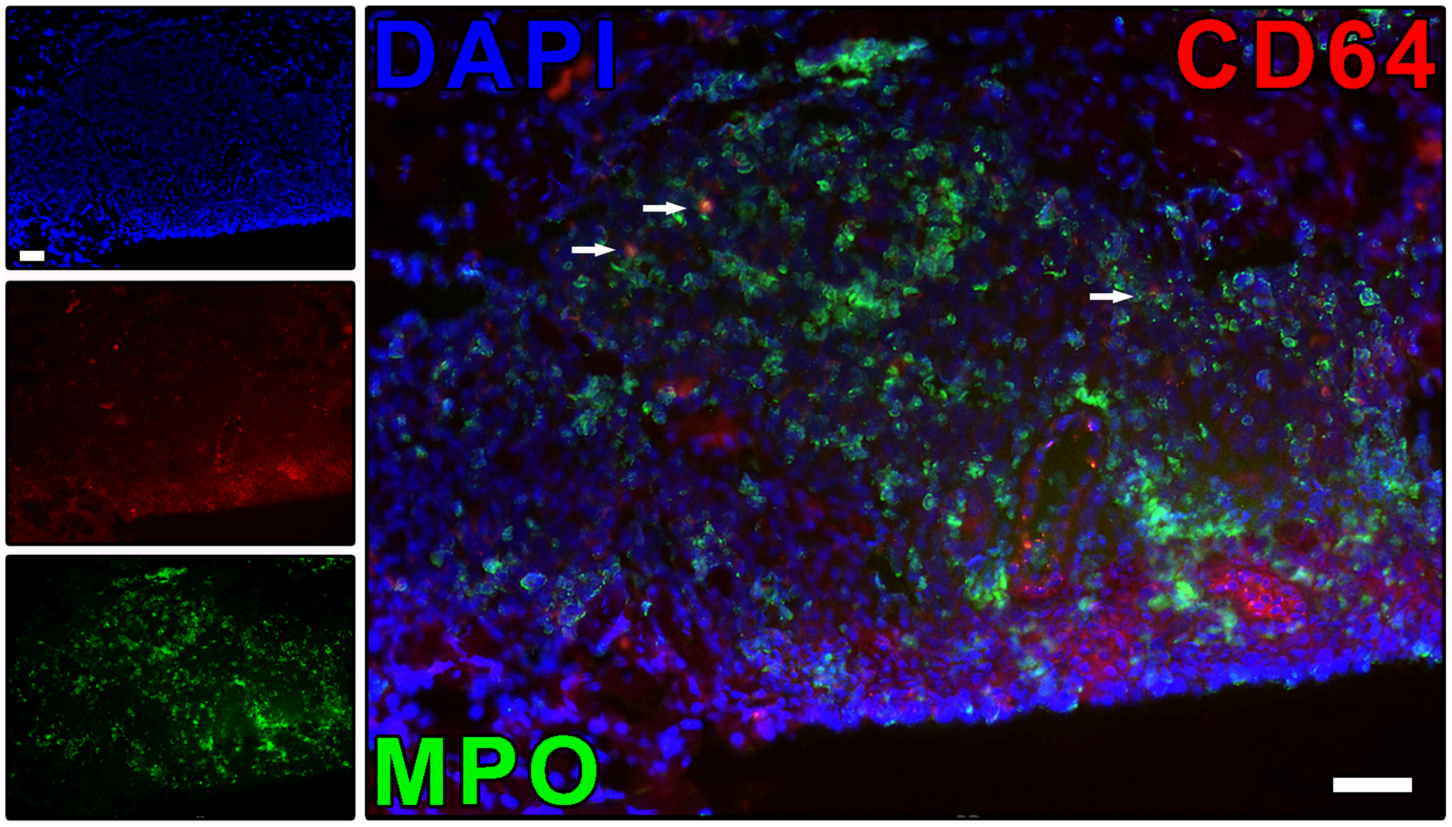
ENL skin lesions present neutrophils expressing CD64. The protein expression of CD64 on neutrophil (MPO^+^ cells) was evaluated by immunofluorescence. Skin lesion was stained for CD64 (red), myeloperoxidase (MPO; green), and nuclei (DAPI; blue). Images are representative of three independent samples of ENL patients. Co-localized areas of MPO^+^CD64^+^ cells were identified with arrows. The regulation of neutrophil CD64 expression has the potential to be useful in the ENL treatment, as well as to prevent ENL reactional episodes. Scale bars: 10 μm. Images from Leprosy Laboratory collection.

Our group has demonstrated that, during ENL, circulating and lesional neutrophils exclusively expresses CD64 (FcγRI) while leprosy patients without reaction, such as BL/LL, BT, and RR individuals, do not (9) (Figure 3). Besides, the higher CD64 levels on circulating neutrophils have been correlated with disease severity, pointing to CD64 as an early biomarker for ENL as well as a marker of severity (9). Since neutrophils function as biosensors, the proinflammatory microenvironment and/or fragments of the bacillus could induce the expression of CD64 on the neutrophil surface. The biological impact of surface expression of CD64 in neutrophils needs to be better evaluated. Upregulation of CD64 *in vivo* has likewise been associated with enhanced neutrophilic functionality (91–93). At the onset of sepsis or septic shock, the CD64 expression rate in neutrophils is augmented (94). Fadlon et al. showed that the CD64^+^ neutrophils bound to endothelial monolayers and CD64^+^enriched neutrophils were 7 times more strongly adherent to endothelial monolayers than were CD64-depleted neutrophils (95).

Pentraxin-3 (PTX3), a protein present in the secondary neutrophilic granule, was originally identified as being induced by such primary inflammatory signals as TNF and interleukin 1β (IL-1β) (96, 97). Our group has shown evidence that PTX3 is released systemically and at the site of ENL lesions (98). Our research has also demonstrated that PTX3 serum levels correlated to the surface expression of CD64 in circulating neutrophils and that thalidomide treatment of ENL down regulated PTX3 levels (98). Interestingly, PTX3 serum levels were high in MB patients without reaction yet persistent in patients who developed ENL. In contrast, MB patients who developed RR had low levels of PTX3 prior to and at the onset of the event. These results indicate that high and persistent levels of PTX3 in MB patients may be associated with the occurrence of ENL while also identifying PTX3 as a potentially predictive ENL biomarker capable of differentiating it from an RR episode.

In recent years, several large-scale gene expression studies have been conducted to monitor the host response to pathogen. These study results could potentially serve as diagnostic tool to either distinguish disease-afflicted patients from healthy individuals or classify different forms of the same disease. Via microarray analyses, Lee et al. (99) compared LL -reaction free skin lesions to those of ENL patients. Their global gene expression profiles revealed the up-regulation of genes involved in cell-movement, including E-selectin and its ligands, both key molecules in mediating neutrophilic recruitment to inflammatory sites (99). Transcriptome profiles derived from ENL skin lesions have also recently detailed the participation of neutrophilic and endothelial cell-gene networks in the vasculitis resulting in tissue damage (100).

To date, no study has yet reported a gene expression signature based on leprosy whole blood. However, via the microarray, the global transcriptional profiles of PBMC revealed that there were 275 genes differentially expressed in RR and 517 differentially expressed in ENL (101). In addition, a granulocytic gene signature was identified in gene-expression arrays derived from ENL PBMCs (101). These data suggest that PBMC fractions of ENL patients may be contaminated with LDN subpopulation, as has been similarly described in autoimmune diseases. Nonetheless, the presence of LDN in light of their functional capacity and potential to contribute to the clinical manifestations of ENL remain basically unexplored.

Naranbhai et al. have recently mapped the quantitative trait loci (eQTL) expression in peripheral blood CD16^+^ neutrophils from 101 healthy Europeans (102). The analyses found that leprosy and Crohn's disease, an autoimmune inflammatory bowel illness, showed a profound overlap in genetic architecture. The ancestral T allele of rs1981760 was associated with an increased susceptibility to MB leprosy. The authors observed a strong link between rs1981760-T and a reduced NOD2 expression in neutrophils in conjunction with a conversely elevated expression in monocytes. In addition, neutrophils stimulated with a NOD2 ligand, muramyl dipeptide supplemented with Pam3-CSK4, a synergistic agonist, express significantly higher levels of mRNA for IFNβ (102). These data demonstrate that rs1981760 affects NOD2 expression and the subsequent IFNβ responses to its ligand. Interestingly, eQTL in neutrophils are enriched for genes in the IFNβ network. These data suggest that type-1 interferons and neutrophils may be involved in leprosy such as has been previously shown in tuberculosis (103).

## Conclusion

There are still large gaps in our understanding of the role of neutrophils in ENL and leprosy disease despite the large number of studies examining their immunological functions. Future works should aim to further determine the roles of neutrophils in host–mycobacterial interactions, particularly as relates to their early defensive posture and possible contribution to disease progression. The identification of subpopulations of neutrophils associated with the clinical forms of leprosy could provide novel insights of neutrophil function and reveal new targets in leprosy. The present review suggests the roles performed by neutrophils as both migratory and, for the first time, effector cells following chemo-attractants in the context of leprosy.

## Author Contributions

VS wrote the original draft of manuscript. IT, FP, JdS, and CdS helped with literature collating and referencing. IT, PP, AM, and ES revised and edited the manuscript.

### Conflict of Interest Statement

The authors declare that the research was conducted in the absence of any commercial or financial relationships that could be construed as a potential conflict of interest.
